# Acute Pancreatitis in Individuals with Sickle Cell Disease: A Systematic Review

**DOI:** 10.3390/jcm13164712

**Published:** 2024-08-11

**Authors:** Chinenye R. Dike, Adefunke DadeMatthews, Oluwagbemiga DadeMatthews, Maisam Abu-El-Haija, Jeffrey Lebensburger, Abigail Smith, Aamer Imdad

**Affiliations:** 1 Department of Pediatrics, Division of Gastroenterology, Hepatology and Nutrition, University of Alabama at Birmingham, Birmingham, AL 35233, USA; 2 Department of Human Development and Family Studies, College of Human Sciences, Auburn University, Auburn, AL 36849, USA; aod0006@auburn.edu; 3 School of Kinesiology, College of Human Sciences and Education, Louisiana State University, Baton Rouge, LA 70803, USA; gdadematthews@lsu.edu; 4 Department of Pediatrics, College of Medicine, University of Cincinnati, Cincinnati, OH 45267, USA; maisam.haija@cchmc.org; 5 Division of Gastroenterology, Hepatology and Nutrition, Cincinnati Children’s Hospital Medical Center, Cincinnati, OH 45229, USA; 6 Department of Pediatrics, Division of Hematology and Oncology, University of Alabama at Birmingham, Birmingham, AL 35233, USA; jlebensburger@uabmc.edu; 7 Health Science Library, Upstate Medical University, Syracuse, NY 13210, USA; smithab@upstate.edu; 8 Stead Family Department of Pediatrics, Division of Gastroenterology, Hepatology, and Nutrition, University of Iowa, Iowa City, IA 52242, USA; aamer-imdad@uiowa.edu

**Keywords:** acute pancreatitis, sickle cell disease, gallstones

## Abstract

**Background/Objectives**: Sickle cell disease (SCD) impacts about 100,000 people in the US. SCD increases the risk of cholelithiasis and microvascular ischemia, which could increase the risk of acute pancreatitis (AP). Abdominal pain is a common presenting symptom of AP and sickle cell vaso-occlusive crisis. The purpose of our systematic review is to estimate the prevalence and determine the severity of AP in individuals with SCD compared to the general population. **Methods**: Multiple electronic databases were searched. We included studies that included children and adults (population) and addressed the association of SCD (exposure) with AP (outcome) compared to the same population without SCD (control). Two authors screened titles and abstracts independently, and data were abstracted in duplication from included studies. We registered this protocol in PROSPERO-CRD42023422397. **Results**: Out of 296 studies screened from multiple electronic databases, we identified 33 studies. These studies included 17 case reports, one case series, and 15 retrospective cohort studies, and 18 studies included children. Eight of the AP case reports were in patients with HbSS genotype, two with sickle beta thalassemia, and one with HbSoArab, and in six case reports, a genotype was not specified. Complications were reported in 11 cases—respiratory complication (in at least four cases), splenic complications (three cases), pancreatic pseudocyst (two cases) and death from AP (one case). Of the four AP cases in the case series, three had HbSS genotype, and two cases had complications and severe pancreatitis. AP prevalence in SCD was estimated to be 2% and 7% in two retrospective studies, but they lacked a comparison group. In retrospective studies that evaluated the etiology of AP in children, biliary disease caused mostly by SCD was present in approximately 12% and 34%, respectively. **Conclusions:** Data on the prevalence of AP in individuals with SCD are limited. Prospectively designed studies aiming to proactively evaluate AP in individuals with SCD who present with abdominal pain are needed to improve timely diagnosis of AP in SCD and outcomes.

## 1. Introduction

Sickle cell disease (SCD) is an inherited blood disorder that affects millions of individuals worldwide and disproportionately affects Black and African American populations [1]. Individuals with SCD have a reduced life expectancy and income loss compared to the general population [2].

Pain or vaso-occlusive crisis (VOC) is the hallmark of the disease and is a cause of increased healthcare utilization and morbidity [3]. Pain locations may vary in individuals with SCD during a VOC [4]. Abdominal pain is a common symptom of VOC but may also be the presenting symptom for many other disorders [5]. Abdominal pain is also a presenting symptom of acute pancreatitis (AP) [6,7]. Individuals with SCD might be at increased risk of AP due to microvascular ischemia and increased incidence of gallstone disease [8,9]. Gallstones are a common etiology of AP in both children and adults [10,11,12].

Despite an increased risk of AP in individuals with SCD, we found that children with SCD who presented to our emergency department (ED) with abdominal pain over a 2-year period were rarely systematically investigated for gastrointestinal (GI) disorders including AP [13]. In our single-center retrospective study, less than 50% of children with SCD and abdominal pain had any GI-specific workup, only a quarter had a lipase check, and only 6% had an amylase check [13].

Given the biological plausibility for increased risk of AP in individuals with SCD, we conducted a systematic review to evaluate the prevalence and severity of AP in patients with SCD.

## 2. Materials and Methods

This systematic review was conducted according to the Cochrane Handbook [14] and is being reported using the Preferred Reporting Items for Systematic Reviews and Meta-Analyses (PRISMA) guidelines 2020 [15]. We developed and registered the protocol for the systematic review in an online database, PROSPERO (CRD42023422397).

### 2.1. Types of Studies

Observational studies included cohort studies and case–control studies. We also included case series and case reports.

### 2.2. Population

The study population included adults and children with SCD with homozygous mutations in the beta globulin gene including those with sickle beta thalassemia such as HbSS, HbSC, Sickle beta 0 thalassemia (Hb S/β0-Thal), and Sickle beta plus thalassemia (HbS β + thal).

### 2.3. Exposure

Acute pancreatitis (AP) was defined using both the adult and pediatric guidelines [6,7], irrespective of the AP etiology. We excluded studies of patients with sickle cell trait and chronic pancreatitis.

### 2.4. Comparison

We compared the study population with adults and children without SCD.

### 2.5. Outcomes

The study outcomes were as follows:

1. AP prevalence (%) in SCD when compared to the general population.
2. AP severity as defined by the adults: RANSON (≥3) [16], APACHE II (≥8) [17], Revised Atlanta criteria (Moderately severe to severe AP) [18]. Pediatric NASPGHAN classification (Moderately severe to severe AP) [19].
3. Length of hospital stay.
4. Need for intensive care admission.
5. Mortality.
6. Presence of local pancreatic and peripancreatic complications (necrosis, hemorrhage, cysts).
7. Need for procedural interventions during index admission.

### 2.6. Literature Search Including Data Extraction

Systematic searches using a combination of keywords and controlled vocabulary (where available) related to SCD and AP were conducted in PubMed, EMBASE, CINAHL, Scopus, Web of Science Core Collection, and WHO Global Index Medicus. All databases were searched from inception to 8 May 2023, with no restrictions on language, publication status, outcomes, or publication year. We also searched abstracts of major gastroenterology conferences. The search strategies are available in online Supplemental Material File S1.

We used a 3-phased approach for data extraction—screening of titles and abstracts, full-text review, and data extraction using at least two authors (A.D. and O.D.) for each of these phases to develop a consensus, with conflicts resolved by authors (C.R.D. and A.I.). Covidence software (extraction 2.0) was used for data screening and extraction [20]. We extracted the data on study population, sample size, criteria for AP diagnosis and etiology of AP, and outcomes of interest.

### 2.7. Data Synthesis

We used the Newcastle–Ottawa Quality Assessment Scale for risk of bias assessments for the observational studies. We had planned to perform a random effects meta-analysis; however, we did not have enough studies to conduct a meta-analysis.

### 2.8. Grading of Quality of Evidence

We used The Grading of Recommendations Assessment, Development and Evaluation (GRADE) to rate the overall certainty of evidence [21].

## 3. Results

**Figure 1:**
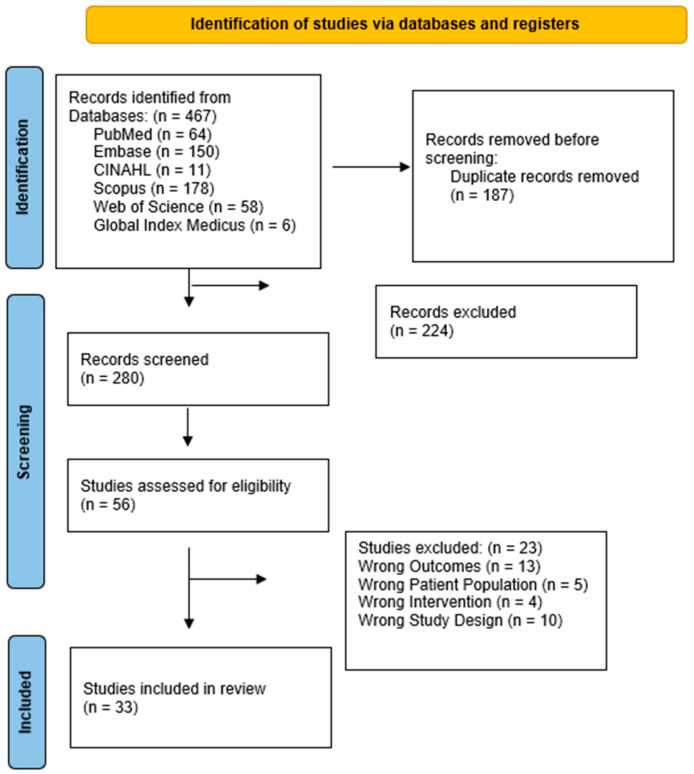
Flow diagram of included studies.

A total of 280 titles and abstracts were screened. Of these, 56 eligible studies were screened for full-text review, and ultimately, 33 studies met the inclusion criteria and were included [22,23,24,25,26,27,28,29,30,31,32,33,34,35,36,37,38,39,40,41,42,43,44,45,46,47,48,49,50,51,52,53,54]. Twenty-three studies were excluded; thirteen studies were excluded for outcomes that did not match the proposed outcomes, four studies were excluded for interventions that did not match the search criteria, five studies were excluded for inappropriate study populations, and one study was excluded for inappropriate study design. Please see the flow chart (Figure 1) for details.

### 3.1. Study Types

Among the 33 studies included, 17 studies were case reports, 1 was a case series of 4 cases, and 15 were retrospective cohort studies. Out of the 15 retrospective cohort studies, 2 studies did not have a full text for review; in this case, only the abstract was reviewed and data from the abstract extracted.

### 3.2. Characteristics of Included Studies

Eighteen out of the thirty-three studies included in this systematic review also included children. Eight of the seventeen AP case reports were in individuals with HbSS genotype, while two were in those with sickle cell beta thalassemia, and one was in a patient with HbSoArab genotype, and in six case reports, the genotype was not specified. One case was reported in a pregnant woman. Three out of the four AP cases reported in the case series were in individuals with HbSS genotype. One child (25%) younger than 18 years old was reported in the case series, and six (35%) of the case reports were in children. Eleven of the case reports were in males (65%), and there were two males (50%) in the case series (Supplemental Table S1). There was inadequate documentation of sex across the observational studies. Only two of the studies reported the prevalence [39,47], and two studies reported the etiology of cases of AP in their cohort that included both individuals with SCD and those without SCD [24,54]. The other studies include those investigating the prevalence and complications of cholelithiasis in an SCD cohort [26,32,33,35], the management of cholelithiasis and choledocholithiasis in children with SCD [25], surgical options for splenectomy in children with SCD [27], biliary tract disease in children and young adults with SCD [28], digestive diseases in SCD [30], the etiology of intensive care admissions in children with SCD [37], indications for endoscopic retrograde cholangiopancreatography in children with SCD [22], and the etiology and complications of *Clostridioides difficile* infection in a hospitalized cohort of individuals with SCD [50] (Supplemental Table S2).

### 3.3. AP Presentation

All the cases reported in the literature had abdominal pain. Additional lab findings and symptoms for these cases are presented in Supplemental Table S1.

### 3.4. Outcomes

#### 3.4.1. AP Prevalence

We could only identify two retrospective cohort studies that estimated the prevalence of AP in SCD, at 2% [47] and 7% [39], respectively. These estimates were based on diagnosis of AP in patients in their cohorts who presented with abdominal pain and were tested for AP. No natural history studies were identified that systematically assessed the prevalence of AP.

#### 3.4.2. AP Complications

We identified 14 patients who experienced complications among the 21 cases of AP in the literature. In the case series, 2 of 4 patients were identified with an acute complication, and 12 of 17 patients from the case reports experienced an acute complication. One patient died due to AP complications. The remaining complications included five cases of respiratory complications, four cases of splenic complications, two cases of ascites, two cases of pancreatic pseudocysts, and one case of renal complication. Among the case reports and case series of patients with SCD and AP, many had complications due to AP—50% (2) of the reported cases in the case series developed complications [23], and nearly 71% (12) of the reported cases in the case reports developed complications. This includes one (8%) reported death due to AP [52]. Other complications reported include respiratory complications in five cases (approximately 42%) [29,42,43,48,53], renal complications in one case (8%) [46], splenic complications in four cases (33%) [31,34,42,51], ascites in two cases (17%) [42,49], and pancreatic pseudocyst in two cases (17%) [36,40].

### 3.5. AP Etiology

The 21 cases of AP were associated with two identified etiologies. In total, eight patients had AP caused by cholelithiasis or choledocholithiasis, including seven patients (41%) from the case reports [41,42,45,46,49,52,53] and one patient (25%) in the case series [23]. Alcohol and polysubstance use was reported in one of the case reports [46]. However, the history was negative for alcohol intake before presentation, and the alcohol breath test was negative [46]. In two retrospective cohort studies evaluating AP etiology in a pediatric cohort, gallbladder disease caused by SCD was reported as 12% [54] and 34% [24], respectively. Further, in one of the retrospective studies that reported prevalence of AP, gallstones accounted for nearly 67% and 71% of AP in adults with HbSC and HbSS genotypes, respectively [39].

### 3.6. Risk of Bias Assessment and Grading of Quality Evidence

The risk of bias assessment rating was poor for the studies evaluating our primary outcome of prevalence, and the certainty of evidence was low for both studies (Table 1 and Table 2).

## 4. Discussion

In this systematic review evaluating the prevalence and severity of AP in both children and adults with SCD, we found that the literature is mostly limited to case reports and a few observational studies. Although we had planned a meta-analysis, we could not perform this due to the lack of studies with similar populations, exposure and comparison groups.

There were two studies that estimated the prevalence of AP in SCD to be 2–7% in children and adults, respectively [39,47], with one of the studies concluding that the prevalence of AP in SCD is similar to that in the general population [39]. Both studies had published abstracts only and did not have full texts available for review. Unfortunately, they were both retrospective studies and only estimated the prevalence of AP in their cohort with SCD. Further, although the pediatrics study had about 500 children in its cohort, out of the 123 admissions who had right upper quadrant pain, only 50% had an amylase level check, of which 16% had elevated amylase levels [47]. We have also shown in our cohort that children with SCD who present to the emergency department with abdominal pain rarely receive gastrointestinal investigations [13]. In our prior study, we found that lipase was obtained in 25%, amylase in 6%, and abdominal ultrasound performed in 16% of children with SCD presenting with abdominal pain. In those checked, about 3%, 6%, and 4% met the definitions of AP, respectively, for lipase, amylase, and abdominal ultrasound. Therefore, the reported prevalence of AP in the SCD patients is likely underestimated.

Cholelithiasis (gallstones) occurs at an increased frequency in individuals with SCD due to chronic hemolysis. Gallstones are a common cause of AP (often called biliary AP) in up to 33–39% of all patients [55,56]. There is a wide range of reported incidence of gallstone disease in individuals with SCD, varying from 3.1% to up to 57%, based on the sickle cell phenotype, age, and geographic location [9,57]. We found that biliary AP (AP caused by gallstones) accounted for 12–34% of cases in children with SCD in two observational studies that evaluated AP etiology in children [24,54]. SCD accounted for nearly 83% of biliary AP in one of the studies [24]. Further, Jasti and colleagues reported a higher rate of biliary AP in their cohort of adults with SCD and AP of 67% and 71% for HbSC and HbSS genotypes, respectively [39].

Hemoglobin SS (HbSS) genotype appeared to be the predominant genotype in the case reports and cohort studies included in this systematic review. In the study estimating the prevalence of AP in SCD [39], nearly 88% of the cases of AP identified in adults with SCD occurred in those with HbSS genotype, while 90% of the identified AP cases in the pediatric SCD study occurred in those with HbSS genotype [47]. Therefore, although the estimated prevalence of AP in SCD may not be accurate given the retrospective study design, AP in individuals with SCD appears to be prevalent in those with HbSS genotype and should be investigated in future prospective studies.

In this systematic review, we found that AP complications and length of stay may be higher in individuals with SCD. Al-Hindi et al. [24] reported in their study that all their patients with SCD and cholelithiasis were admitted to the intensive care unit (ICU) out of an abundance of caution but ended up with at least an additional 2-day stay in the ICU compared to other patients. In the pediatric study estimating AP prevalence in SCD, children with SCD who had pancreatitis had the longest hospitalization stay of 7–38 days (mean = 19.8 days) when compared to those with sickle cell hepatopathy (mean = 7.2 days) or cholelithiasis (mean = 4.6 days) alone [47]. These data, however, are based on an SCD population with a comparison to the general population, limiting the inference about increased risk of complications related to AP in patients with SCD.

Even though there are descriptive reviews on the association of SCD with AP, this is the first attempt to systematically review the literature on this subject. A limitation of this systematic review is the limited number of studies estimating the prevalence and severity of AP in individuals with SCD, indicating the gaps in knowledge in this area. Future studies should follow patients prospectively with a control group without SCD. It is also important to establish the etiology of pancreatitis in patients with SCD and assess whether these patients are at higher risk of gallstone-related AP compared to a general population with gallstones. Future studies should also determine the risk factors for AP in patients with SCD because there might be unique risk factors such as increased incidence of gallstones disease and ischemic injury due to repeated sickle cell crises. Finally, studies should assess the possibility of chronic pancreatitis in patients with SCD with recurrent abdominal pain.

## 5. Conclusions

In summary, our systematic review shows a paucity of studies on the prevalence of AP in SCD. AP in SCD appears to be more prevalent in those with HbSS genotype. Given the increased risk of gallstones in patients in SCD, well-designed prospective studies estimating the prevalence and severity of AP in individuals with SCD should be conducted.

## Figures and Tables

**Table 1:** Risk of bias assessment of studies reporting prevalence of acute pancreatitis in sickle cell disease.

Population: Adult and Pediatric Exposure: Sickle Cell Disease Comparison: No Sickle Cell Disease			
Outcome	Number of Studies and Effect Size	Certainty of Evidence	Comments
Acute Pancreatitis Prevalence	Studies: 2 Reported Prevalence: 2% and 7%	Very Low	Two of the included studies were small and had poor quality based on the Ottawa Scale. No meta-analysis was conducted due to lack of homogenous studies. There was no comparison group.

**Table 2:** Rating of evidence of studies reporting prevalence of acute pancreatitis in sickle cell disease using the Ottawa Scale.

Study	Rating Based on Ottawa Scale	Comments
Jasti 2008 [39]	Poor	The study population might not have been representative because the patients were recruited from a single institution. There was no comparison group. SCD, however, was defined with electrophoresis and the definition of outcome (AP) was clearly described, but it was not a blinded assessment.
Sakhalkar 2004 [47]	Poor	The study population might not have been representative because the patients were recruited from a single institution. There was no comparison group. SCD was defined, and specific genotypes were noted. The definition of outcome (AP) was not clearly described, and it was not a blinded assessment.

## Data Availability

All data used for the systematic review are included in Supplemental Tables S1 and S2 and Supplemental Material File S1.

## Supplementary Materials

The following supporting information can be downloaded at: https://www.mdpi.com/article/10.3390/jcm13164712/s1, Supplemental Table S1: Summary of included case reports and case series; Supplemental Table S2: Summary of included observational studies; Supplemental Material File S1: Search strategy used.
