# Supplementary material for: Acute Pancreatitis in Individuals with Sickle Cell Disease: A Systematic Review
Source: J Clin Med. 2024 Aug 11;13(16):4712. doi: 10.3390/jcm13164712 (PMC11355684; doi:10.3390/jcm13164712)
Supplement: Supplementary file 1 [file jcm-13-04712-s001.zip › Supplemental Material File S1_search strategy.pdf]

Supplemental Material File S1

**Appendix 1**

**All Searches were completed on May 8, 2023**

**PubMed**

("Pancreatitis"[MeSH Terms] OR ("Pancreatitis"[Title/Abstract] OR "Pancreatitides"[Title/Abstract] OR "acute pancreas"[Title/Abstract:~3] OR "acute pancreatic"[Title/Abstract:~3] OR "inflamed pancreas"[Title/Abstract:~3] OR "inflamed pancreatic"[Title/Abstract:~3] OR "pancreatic inflammation"[Title/Abstract:~3] OR "pancreas inflammation"[Title/Abstract:~3] OR "pancreatic edema"[Title/Abstract:~3] OR "pancreatic oedema"[Title/Abstract:~3] OR "pancreas edema"[Title/Abstract:~3] OR "pancreas oedema"[Title/Abstract:~3] OR "pancreatic necrosis"[Title/Abstract:~3] OR "pancreatic necroses"[Title/Abstract:~3] OR "necrotic pancreatic"[Title/Abstract:~3] OR "necrotize pancreatic"[Title/Abstract:~3] OR "necrotise pancreatic"[Title/Abstract:~3] OR "necrotizing pancreatic"[Title/Abstract:~3] OR "necrotising pancreatic"[Title/Abstract:~3] OR "necrotized pancreatic"[Title/Abstract:~3] OR "necrotised pancreatic"[Title/Abstract:~3]  OR "pancreas necrosis"[Title/Abstract:~3] OR "pancreas necroses"[Title/Abstract:~3] OR "necrotic pancreas"[Title/Abstract:~3] OR "necrotize pancreas"[Title/Abstract:~3] OR "necrotise pancreas"[Title/Abstract:~3] OR "necrotizing pancreas"[Title/Abstract:~3] OR "necrotising pancreas"[Title/Abstract:~3] OR "necrotized pancreas"[Title/Abstract:~3] OR "necrotised pancreas"[Title/Abstract:~3] OR "Hemorrhagic Pancreas"[Title/Abstract:~3] OR "Haemorrhagic Pancreas"[Title/Abstract:~3] OR "Hemorrhaging Pancreas"[Title/Abstract:~3] OR "Haemorrhaging Pancreas"[Title/Abstract:~3] OR "Hemorrhage Pancreas"[Title/Abstract:~3] OR "Haemorrhage Pancreas"[Title/Abstract:~3] OR "Hemorrhaged Pancreas"[Title/Abstract:~3] OR "Haemorrhaged Pancreas"[Title/Abstract:~3] OR "Pancreas Hemorrhages"[Title/Abstract:~3] OR "Pancreas Haemorrhages"[Title/Abstract:~3] OR "Hemorrhagic Pancreatic"[Title/Abstract:~3] OR "Haemorrhagic Pancreatic"[Title/Abstract:~3]  OR "Hemorrhaging Pancreatic"[Title/Abstract:~3] OR "Haemorrhaging Pancreatic"[Title/Abstract:~3] OR "Pancreatic Hemorrhage"[Title/Abstract:~3] OR "Pancreatic Haemorrhage"[Title/Abstract:~3]  OR "Hemorrhaged Pancreatic"[Title/Abstract:~3] OR "Haemorrhaged Pancreatic"[Title/Abstract:~3]  OR "Pancreatic Hemorrhages"[Title/Abstract:~3] OR "Pancreatic Haemorrhages"[Title/Abstract:~3])) AND ("anemia, sickle cell"[MeSH Terms] OR ("sickle cell anemia*"[Title/Abstract] OR "sickle cell anaemia*"[Title/Abstract] OR "sickle cell disease*"[Title/Abstract] OR "drepanocytemia*"[Title/Abstract] OR "drepanocytic anaemia*"[Title/Abstract] OR "drepanocytic anemia*"[Title/Abstract] OR "drepanocytosis"[Title/Abstract] OR "haemoglobin SS"[Title/Abstract] OR "hemoglobin SS"[Title/Abstract] OR "hb ss disease*"[Title/Abstract] OR "hbss disease*"[Title/Abstract] OR "hemoglobin s disease*"[Title/Abstract] OR "haemoglobin s disease*"[Title/Abstract] OR "hbs disease*"[Title/Abstract] OR "hb s

disease*"[Title/Abstract] OR "homozygous sickle cell anaemia*"[Title/Abstract] OR "homozygous sickle cell anemia*"[Title/Abstract] OR "homozygous sickle cell disease*"[Title/Abstract] OR "meniscocytosis"[Title/Abstract] OR "sickle anaemia*"[Title/Abstract] OR "sickle anemia*"[Title/Abstract] OR "ss disease*"[Title/Abstract]))

**Embase**

**#9** #5 AND #8

**#8** #6 OR #7

**#7** 'sickle cell anemia*':ti,ab OR 'sickle cell anaemia*':ti,ab OR 'sickle cell disease*':ti,ab OR 'drepanocytemia*':ti,ab OR 'drepanocytic anaemia*':ti,ab OR 'drepanocytic anemia*':ti,ab OR 'drepanocytosis':ti,ab OR 'haemoglobin ss':ti,ab OR 'hemoglobin ss':ti,ab OR 'hb ss disease*':ti,ab OR 'hbss disease*':ti,ab OR 'hemoglobin s disease*':ti,ab OR 'haemoglobin s disease*':ti,ab OR 'hbs disease*':ti,ab OR 'hb s disease*':ti,ab OR 'homozygous sickle cell anaemia*':ti,ab OR 'homozygous sickle cell anemia*':ti,ab OR 'homozygous sickle cell disease*':ti,ab OR 'meniscocytosis':ti,ab OR 'sickle anaemia*':ti,ab OR 'sickle anemia*':ti,ab OR 'ss disease*':ti,ab

**#6** 'sickle cell anemia'/exp

**#5** #1 OR #2 OR #3 OR #4

**#4** pancreatitis:ti,ab OR pancreatitides:ti,ab OR ((acute NEAR/3 pancreas):ti,ab) OR ((acute NEAR/3 pancreatic):ti,ab) OR ((inflamed NEAR/3 pancreas):ti,ab) OR ((inflamed NEAR/3 pancreatic):ti,ab) OR ((pancreatic NEAR/3 inflammation):ti,ab) OR ((pancreas NEAR/3 inflammation):ti,ab) OR ((pancreatic NEAR/3 edema):ti,ab) OR ((pancreatic NEAR/3 oedema):ti,ab) OR ((pancreas NEAR/3 edema):ti,ab) OR ((pancreas NEAR/3 oedema):ti,ab) OR ((pancreatic NEAR/3 necros?s):ti,ab) OR ((necrotic NEAR/3 pancreatic):ti,ab) OR ((necroti?e* NEAR/3 pancreatic):ti,ab) OR ((necroti?ing NEAR/3 pancreatic):ti,ab) OR ((pancreas NEAR/3 necros?s):ti,ab) OR ((necrotic NEAR/3 pancreas):ti,ab) OR ((necroti?e* NEAR/3 pancreas):ti,ab) OR ((necroti?ing NEAR/3 pancreas):ti,ab) OR ((hemorrhag* NEAR/3 pancreas):ti,ab) OR ((haemorrhag* NEAR/3 pancreas):ti,ab) OR ((hemorrhag* NEAR/3 pancreatic):ti,ab) OR ((haemorrhag* NEAR/3 pancreatic):ti,ab)

**#3** 'hemorrhagic pancreatitis'/exp

**#2** 'acute hemorrhagic pancreatitis'/exp

**#1** 'acute pancreatitis'/exp

**CINAHL**

**S9** S4 AND S8

**S8** S5 OR S6 OR S7

**S7** AB ("sickle cell an#emia*" OR "sickle cell disease*" OR "drepanocytemia*" OR "drepanocytic an#emia*" OR "drepanocytosis" OR "h#emoglobin SS" OR "hb ss disease*" OR "hbss disease*" OR "h#emoglobin s disease*" OR "hbs disease*" OR "hb s disease*" OR "homozygous sickle cell an#emia*" OR "homozygous sickle cell disease*" OR "meniscocytosis" OR "sickle an#emia*" OR "ss disease*")

**S6** TI ("sickle cell an#emia*" OR "sickle cell disease*" OR "drepanocytemia*" OR "drepanocytic an#emia*" OR "drepanocytosis" OR "h#emoglobin SS" OR "hb ss disease*" OR "hbss disease*" OR "h#emoglobin s disease*" OR "hbs disease*" OR "hb s disease*" OR "homozygous sickle cell an#emia*" OR "homozygous sickle cell disease*" OR "meniscocytosis" OR "sickle an#emia*" OR "ss disease*")

**S5** (MH "Anemia, Sickle Cell+")

**S4** S1 OR S2 OR S3

**S3** AB (pancreatitis OR pancreatitides OR acute N3 pancreas OR acute N3 pancreatic OR inflamed N3 pancreas OR inflamed N3 pancreatic OR pancreatic N3 inflammation OR pancreas N3 inflammation OR pancreatic N3 edema OR pancreatic N3 oedema OR pancreas N3 edema OR pancreas N3 oedema OR pancreatic N3 necros#s OR necrotic N3 pancreatic OR necroti#e* N3 pancreatic OR necroti#ing N3 pancreatic OR pancreas N3 necros#s OR necrotic N3 pancreas OR necroti#e* N3 pancreas OR necroti#ing N3 pancreas OR h#emorrhag* N3 pancreas OR h#emorrhag* N3 pancreatic)

**S2** TI (pancreatitis OR pancreatitides OR acute N3 pancreas OR acute N3 pancreatic OR inflamed N3 pancreas OR inflamed N3 pancreatic OR pancreatic N3 inflammation OR pancreas N3 inflammation OR pancreatic N3 edema OR pancreatic N3 oedema OR pancreas N3 edema OR pancreas N3 oedema OR pancreatic N3 necros#s OR necrotic N3 pancreatic OR necroti#e* N3 pancreatic OR necroti#ing N3 pancreatic OR pancreas N3 necros#s OR necrotic N3 pancreas OR necroti#e* N3 pancreas OR necroti#ing N3 pancreas OR h#emorrhag* N3 pancreas OR h#emorrhag* N3 pancreatic)

**S1** (MH "Pancreatitis+")

**Scopus**

(TITLE-ABS-KEY(pancreatitis OR pancreatitides OR ( acute W/3 pancreas ) OR ( acute W/3 pancreatic ) OR ( inflamed W/3 pancreas ) OR ( inflamed W/3 pancreatic ) OR ( pancreatic W/3 inflammation ) OR ( pancreas W/3 inflammation ) OR ( pancreatic W/3 *edema ) OR ( pancreas W/3 *edema ) OR ( pancreatic W/3 necros?s ) OR ( necrotic

W/3 pancreatic ) OR ( necroti*e* W/3 pancreatic ) OR ( necroti?ing W/3 pancreatic ) OR ( pancreas W/3 necros?s ) OR ( necrotic W/3 pancreas ) OR ( necroti*e* W/3 pancreas ) OR ( necrotizing W/3 pancreas ) OR ( h*emorrhag* W/3 pancreas ) OR ( h*emorrhag* W/3 pancreatic ))) AND (TITLE-ABS-KEY("sickle cell an*emia*" OR "sickle cell disease*" OR drepanocytemia* OR "drepanocytic an*emia*" OR drepanocytosis OR "h*emoglobin SS" OR "hb ss disease*" OR "hbss disease*" OR "h*emoglobin s disease*" OR "hbs disease*" OR "hb s disease*" OR "homozygous sickle cell an*emia*" OR "homozygous sickle cell disease*" OR meniscocytosis OR "sickle an*emia*" OR "ss disease*"))

**Web of Science (SCI-Expanded; ESCI; CPCI-S; SSCI)**

**3** #1 AND #2

**2** TS=("sickle cell an$emia*" OR "sickle cell disease*" OR "drepanocytemia*" OR "drepanocytic an$emia*" OR "drepanocytosis" OR "h$emoglobin SS" OR "hb ss disease*" OR "hbss disease*" OR "h$emoglobin s disease*" OR "hbs disease*" OR "hb s disease*" OR "homozygous sickle cell an$emia*" OR "homozygous sickle cell disease*" OR "meniscocytosis" OR "sickle an$emia*" OR "ss disease*")

**1** TS=(pancreatitis OR pancreatitides OR (acute NEAR/3 pancreas )OR (acute NEAR/3 pancreatic) OR (inflamed NEAR/3 pancreas) OR (inflamed NEAR/3 pancreatic) OR (pancreatic NEAR/3 inflammation) OR (pancreas NEAR/3 inflammation) OR (pancreatic NEAR/3 $edema )OR (pancreas NEAR/3 $edema) OR (pancreatic NEAR/3 necros?s) OR (necrotic NEAR/3 pancreatic) OR (necroti?e* NEAR/3 pancreatic) OR (necroti?ing NEAR/3 pancreatic) OR (pancreas NEAR/3 necros?s) OR (necrotic NEAR/3 pancreas) OR (necroti?e* NEAR/3 pancreas) OR (necroti?ing NEAR/3 pancreas) OR (h$emorrhag* NEAR/3 pancreas) OR (h$emorrhag* NEAR/3 pancreatic))

**Global Index Medicus (AIM, IMEMR, IMSEAR, LILACS, WPRO)**

(tw:("Pancreatitis" OR "Pancreatitides" OR "acute pancreas" OR "acute pancreatic" OR "inflamed pancreas" OR "inflamed pancreatic" OR "pancreatic inflammation" OR "pancreas inflammation" OR "pancreatic edema" OR "pancreatic oedema" OR "pancreas edema" OR "pancreas oedema" OR "pancreatic necrosis" OR "pancreatic necroses" OR "necrotic pancreatic" OR "necrotize pancreatic" OR "necrotise pancreatic" OR "necrotizing pancreatic" OR "necrotising pancreatic" OR "necrotized pancreatic" OR "necrotised pancreatic" OR "pancreas necrosis" OR "pancreas necroses" OR "necrotic pancreas" OR "necrotize pancreas" OR "necrotise pancreas" OR "necrotizing pancreas" OR "necrotising pancreas" OR "necrotized pancreas" OR "necrotised pancreas" OR "Hemorrhagic Pancreas" OR "Haemorrhagic Pancreas" OR "Hemorrhaging Pancreas" OR "Haemorrhaging Pancreas" OR "Hemorrhage Pancreas" OR "Haemorrhage Pancreas" OR "Hemorrhaged Pancreas" OR "Haemorrhaged Pancreas" OR "Pancreas Hemorrhages" OR "Pancreas Haemorrhages" OR "Hemorrhagic Pancreatic" OR "Haemorrhagic Pancreatic" OR "Hemorrhaging

Pancreatic" OR "Haemorrhaging Pancreatic" OR "Pancreatic Hemorrhage" OR "Pancreatic Haemorrhage" OR "Hemorrhaged Pancreatic" OR "Haemorrhaged Pancreatic" OR "Pancreatic Hemorrhages" OR "Pancreatic Haemorrhages")) AND (tw:("sickle cell anemia*" OR "sickle cell anaemia*" OR "sickle cell disease*" OR "drepanocytemia*" OR "drepanocytic anaemia*" OR "drepanocytic anemia*" OR "drepanocytosis" OR "haemoglobin SS" OR "hemoglobin SS" OR "hb ss disease*" OR "hbss disease*" OR "hemoglobin s disease*" OR "haemoglobin s disease*" OR "hbs disease*" OR "hb s disease*" OR "homozygous sickle cell anaemia*" OR "homozygous sickle cell anemia*" OR "homozygous sickle cell disease*" OR "meniscocytosis" OR "sickle anaemia*" OR "sickle anemia*" OR "ss disease*"))
