# Supplementary material for: Acute Pancreatitis in Individuals with Sickle Cell Disease: A Systematic Review
Source: J Clin Med. 2024 Aug 11;13(16):4712. doi: 10.3390/jcm13164712 (PMC11355684; doi:10.3390/jcm13164712)
Supplement: Supplementary file 1 [file jcm-13-04712-s001.zip › Supplemental Table S1í¬Case reports new.pdf]

Supplemental Table S1

	Study Name	Patient Characteristics	Sickle Cell Diagnosis/ Clinical Data	Outcomes	Notes
	First Author & Year	Age in years (sex – M/F), comorbidities including biliary disease, Race/Ethnic group	Type of Sickle Cell, Reported Clinical Symptoms, and Labs	Mild vs. Moderately severe to Severe Pancreatitis Complications	Any notes on treatment?
1	Ahmed 2003 (CS)	Case 1: 19(M), Presented with respiratory symptoms (Pneumonia)- Has history of cholecystectomy, no alcohol intake.<br><br>Case 2: 28(F), Metabolic acidosis	HbSS<br><br>Pallor<br><br>Fever (102°F)<br><br>Upper Abdominal Pain<br><br>WBC – 22.7 x 10^9/L<br><br>Amylase – 169 U/l<br><br>Lipase – 798 U/l<br><br>HbSS<br><br>Upper Abdominal Pain<br><br>WBC – 27.1 x 10^9/L<br><br>Amylase – 3799 U/l	Case 1: Mild, No complications<br><br>Case 2: Severe, Pleural effusion, Ascites	Case 1: IVF, Bowel rest, & Transfusion<br><br>Case 2: IVF, Antibiotics, TPN, Transfusion. Required intubation

		Case 3: 20(F), Gall stones,<br>.<br><br>Case 4: 4(M)<br>All African Americans, 3 adults and 1 child (4-year-old male)	Lipase – 841 U/l<br><br>HbSC<br><br>Upper Abdominal Pain<br>Hb – 11.6 g/dl<br>WBC – 23.6 x 10^9/L<br>Amylase – 631 U/l<br>Lipase – 6150 U/l<br><br>HbSS<br>Upper Abdominal Pain<br>Hb – 9.6 g/dl<br>WBC – 20 x 10^9/L<br>Amylase – 258 U/l<br>Lipase – 1757 U/l	Case 3: Severe, Respiratory distress, Splenic infarct, Anemia, Ascites<br>Case 4: Mild, No complications	Case 3: IVF, Hydromorphone, Antibiotics, Transfusion, TPN, Laparoscopic cholecystectomy<br><br>Case 4: IVF, Bowel rest, & Transfusion
2	Badurdeen 2014 (CR)	25(F), Pregnant, SCD Hepathopathy, African American<br>2nd trimester (22 weeks)	HbSS<br><br>Periumbilical Pain<br>Pallor<br>Amylase – 770 U/l<br>Lipase – 926 U/l	Not reported<br>Hypoxia (O2 sat of 76% on RA)	Pancreatitis secondary to ischemia from sickling episodes<br><br>Folic acid & Analgesics

3	Barkin 1995 (CR)	17(M)<br>Race/Ethnic group<br>(study location - USA)	Sickle Thalassemia<br><br>Epigastric pain<br>Fever<br>Hb – 6.5 g/dl<br>Amylase – 161 U/l<br>Lipase – 489 U/l	Splenic infarct	IVF, Analgesics, Antibiotics, Anxiolytics, Psychobehavioral issues related to pain management addressed
4	Kumar 2020 (CR)	8(F), Cholelithiasis<br>Asian (Indian)	Not specified<br>SCD diagnosis made at 9 months old with Hb electrophoresis<br><br>Epigastric and Left Hypochondriac Pain<br>No Fever (99°F)<br>Hb – 8.6 g/dl<br>WBC – 18.1 x 10^9/L<br>Amylase – 459 U/l<br>Lipase – 144 U/l	Small stone in gall bladder and enlarged pancreatic head on US<br><br>CECT- acute edematous pancreas with sludge in gallbladder	IVF, Analgesics, PPIs, Antiemetics, Antibiotics, Hydroxyurea, Folic acid, Octreotide, Transfusion
5	Garikapati 2020 (CR)	40(M),<br>Asian (Indian)	Not specified	Pancreatic pseudocyst	CT-guided pseudocyst drainage via

		No history of alcohol intake or smoking	Upper Abdominal Pain<br>Fever (101.7°F)<br>Hb – 7.6 g/dl<br>WBC – 9.6 x 10^9/L<br>Amylase – 120 U/l<br>Lipase – 100 U/l	No complications reported	percutaneous pigtail catheter<br>No recurrence of cyst post-drainage
6	Hanafy 2020 (CR)	7(M), Arab (Saudi Arabian)<br>CT scan showed edematous pancreas with peripancreatic and retroperitoneal fluid	HbSS<br>Abdominal Pain<br>No fever (99.3°F)<br>Hb – 9.3 g/dl<br>WBC – 14.6 x 10^9/L<br>Amylase – 1016 U/l<br>Lipase – 8414 U/l	Mild splenomegaly	NPO, IVF, Antibiotics, EBT(exchange blood transfusion)<br>ICU out of caution for the first day
7	Hussain 2019 (CR)	19(F), Cholecystectomy at age 11,<br>(study location – USA)	HbSS<br>Epigastric Pain<br>No Fever	No complications reported	IVF, Analgesics, Clear fluid diet

		Hx of multiple admissions for abdominal pain due to VOC<br><br>Presented with epigastric pain radiating to the back.<br><br>Lipase: ~ 24 x ULN<br><br>CT scan: peripancreatic inflammation	Hb – Normal<br><br>WBC – Normal<br><br>Lipase – 1468 U/l		
8	Kumar 1989 (CR)	51(M), Cholecystectomy & Appendectomy at age 43 (8 years prior),<br><br>African American<br><br>No history of smoking or alcohol intake<br><br>Amylase: 2,650<br><br>CT scan showed an edematous pancreas involving the body and tail and pseudocyst in the tail measuring 4cm	Sickle cell hemoglobin 0 disease (SoArab)<br><br>Abdominal Pain<br><br>No Fever<br><br>Hb – 9.3 g/dl<br><br>WBC – 14.5 x 10^9/L<br><br>Amylase – 2650 U/l	Pancreatic pseudocyst	Analgesics, IVF, Blood transfusion, & TPN<br><br>No recurrence of pancreatitis during 12 follow-up period

9	Mehrabani 2019 (CR)	14(M),<br><br>Arab (Iranian)<br><br>Diagnosed at age 6 months via Hb electrophoresis<br><br>US showed cholelithiasis on admission	Not specified<br><br>SCA<br><br>Epigastric and Left Upper Quadrant Abdominal Pain<br><br>No fever (99.7°F)<br><br>Hb – 7.2 g/dl<br><br>WBC – 21.2 x 10^9/L<br><br>Amylase – 1016 U/l<br><br>Lipase – 395 U/l<br><br>Amylase - Normal	Cholelithiasis, Splenomegaly (splenic abscess), Splenic infarct<br><br>Ascites<br><br>Pleural effusion	Analgesics, IVF, oral nutrition as tolerated initially, Antibiotics, Blood transfusion, TPN, Open cholecystectomy (post patient stabilization) & splenectomy due to splenic abscess
10	Moori 2018 (CR)	35(F), Cholelithiasis,<br><br>Ethnicity – NA (study location UK)	Not specified<br><br>Right Upper Quadrant Abdominal Pain<br><br>No fever (99.9°F)<br><br>Hb – 10.8 g/dl<br><br>WBC – 16.9 x 10^9/L<br><br>Amylase – 3308 U/l	Bilateral pleural effusion<br><br>3 day ICU stay	IVF, Antibiotics, Analgesics, Oxygen, , Laparoscopic Cholecystectomy

11	Pasquier 1991 (CR)	11(M), Cholecystectomy at age 6<br>French	HbSS<br><br>Right Hypochondriac Pain<br>No Fever<br>Hb – 7.2 g/dl	Inflamed pancreatic duct (canal of Wirsung)<br>Edema of the pancreas	
12	Popat 2022 (CR)	18(M),<br>Asian (Indian)	HbSS<br><br>Upper Right Quadrant Abdominal Pain<br>No Fever<br>Hb – 8.2 g/dl<br>WBC – 8.2 x 10^9/L<br>Amylase – 1672 U/l<br>Lipase – 7954 U/l	Cholelithiasis, VOC<br>Stones (calclification in pancreatic dct and biliary tree	Blood transfusion, IVF, Antibiotics, Analgesics, Hydroxyurea, Folic acid.<br><br>ERCP-guided pancreatic duct stenting and common bile duct stent inserted
13	Sack 2016 (CR)	48(M), Hx of alcohol intake, Polysubstance abuse (on Methadone)<br><br>Ethnicity – NA (study location – USA)<br><br>He denied alcohol intake prior to	HbSS<br><br>Epigastric Abdominal Pain<br>Lipase – 488 U/l	Retinopathy, CKD stage IV, Cholelithiasis	Drug-induced pancreatitis was ruled out.<br><br>IVF, Blood transfusions, Analgesics, Bowel rest

		admission and had negative alcohol breath test on admission			
14	Shah 2013 (CR)	33(M), Cholecystectomy at age 21,<br><br>African American	Not specified<br><br>Abdominal Pain<br><br>Hb – 5.4 g/dl<br><br>WBC – 11.6 x 10^9/L<br><br>Amylase – 1185 U/l<br><br>Lipase – 1538 U/l	Hypotension, Metabolic acidosis, Respiratory distress necessitating ICU care	Blood transfusions (x 8 units), Supportive therapy
15	Sharma 2023 (CR)	33(F), Hx of Cholelithiasis,<br><br>Asian (Nepalese)	HbSS<br><br>High-performance liquid chromatography<br><br>Epigastric Abdominal Pain<br><br>Hb – 10.6 g/dl<br><br>WBC – 4.3 x 10^9/L<br><br>Amylase – 1847 U/l<br><br>Lipase – 3373 U/l	Ascites	IVF, TPN, Analgesics, Blood transfusions, Laparoscopic Cholecystectomy

16	Sheehan 1993 (CR)	3(F)<br><br>Black (study location – Canada)	Not specified<br><br>Diagnosed at 9 months with SCD via Hb electrophoresis.<br><br>Abdominal Pain<br>No Fever (98.3°F)<br>Hb – 7.6 g/dl<br>WBC – 13.9 x 10^9/L<br>Amylase – 479 U/l<br>Lipase – 5477 U/l	Splenic infarct, Vaso-occlusive disease of right wrist and proximal right tibia	IVF, Analgesics, Oral fluids as tolerated initially, TPN
17	Turnbull 2018 (CR)	37(M), Autoimmune hemolytic anemia, Obese<br><br>Black (Study location – USA)	Sickle beta 0 thalassemia (Hb S/β0-Thal)<br><br>Abdominal Pain<br>No Fever<br>Hb – 8.9 g/dl<br>WBC – 36.9 x 10^9/L<br>Amylase – Unavailable	Hepatomegaly, Cholelithiasis, Severe bradycardia, hypotension, transaminitis, worsening metabolic acidosis, and death.	ICU management for shock, Nasogastric tube drained dark-red blood,<br><br>Autopsy demonstrated Acute pancreatitis<br><br>Cause of death: Acute necrotizing pancreatitis due to

			Lipase – 22 U/l (Normal)		cholelithiasis secondary to SCD
18	Vicari 2008 (CR)	27(M), Cholecystectomy at age 13,	HbSS<br><br>Upper Abdominal Pain<br>Hb – 7.6 g/dl<br>Amylase – 640 U/l	Hepatomegaly, Choledocholithiasis,<br><br>Post-surgical complications - Left bronchopneumonia, Acute chest syndrome, Sepsis, Multiorgan failure, and Death	Choledocholithotomy of multiple pigment<br>stones combined with T-tube drainage was performed

Key:

IVF – Intravenous fluids, TPN – Total parenteral nutrition, PPI – Proton-pump inhibitors, CT – Computerized tomography, EBT – Exchange blood transfusion, MRCP – Magnetic resonance cholangio-pancreatography, VOC – Vaso-occlusive crisis, CKD – Chronic kidney disease
