# Supplementary material for: Acute Pancreatitis in Individuals with Sickle Cell Disease: A Systematic Review
Source: J Clin Med. 2024 Aug 11;13(16):4712. doi: 10.3390/jcm13164712 (PMC11355684; doi:10.3390/jcm13164712)
Supplement: Supplementary file 1 [file jcm-13-04712-s001.zip › Supplemental Table S2í¬Observational studies new.pdf]

Supplemental Table S2

	Study Name	Study Type	Population	Sickle Cell Diagnosis	Outcomes	Notes, Limitations of the study
	First Author & Year		Age range,<br>Patient characteristics<br>Sample size<br>Sex,<br>Ethnicity		% of all pancreatitis caused by Sickle cell<br>% Mild vs. Severe Pancreatitis Complications<br>Describe in the way data was extracted and give percentages and not texts	
1	Al Hindi 2021	Cross sectional	Ages 0-12 with Acute Pancreatitis<br>N=56 (F-41.1%; M-58.9%)<br>Ethnicity Asian (Bahrain)<br><br>69.6% (39) were 6-10 years<br>19.6% (11): 11-12 years<br>10.7% : </=5 years<br>Complications occurred in the 6-10 years range (p=0.013)	Unspecified SCD type	Etiology:<br>Biliary 41.1% (23) (SCD-related 33.9% (19)); Idiopathic 23.2%; Traumatic 19.6%; Drug-induced 8.93%, CF 1.79% (1), pancreatic anomaly 1.79% (1)<br><br>Severity: NA<br><br>Complications:<br>Local: 32.1% (18)<br>[pseudocysts 5.4%(3); cholangitis	Small sample size<br>Retrospective<br>Ultrasound imaging only<br>Missing data on many systemic complications<br>Limited age-based cross comparison<br><br>Mean age: 8.46 years, Average LOS: 7.68 days.<br>All patients had an US- 100%<br>Recurrences 6 (10.7%). Abdominal pain occurred in 100% (56) of patients, vomiting:<br>57.1% (32): fever

					3.6% (2), edema 23.2% (13)]<br>ICU admissions: 41.1% (23)<br>Mortality: 0	35.7% (20), nausea 23.3% (13)<br>Leukocytosis: 35.7% (20)<br>CRP: 8.93% (5)<br>Serum amylase: 80.4% (45)<br>Urinary amylase: 100% (56)<br>Leukocytosis correlated with ICU admissions
2	Almudaibigh 2021	Cross sectional	Ages 0-14 with SCD<br>N=277 (F-45.8%; M-54.2%)<br>Ethnicity - Arab (Saudi-Arabia	HbSS - 69.3%<br>HbSC -0.4%<br>Hb S/β0-Thal- 28.5%<br>Hb S/β+-Thal - 1.8%	Etiology: Not specified<br>Prevalence of cholelithiasis – 31.4%<br>Severity: NA<br>Complications: Total - 29.9%(26)<br>Acute calcular cholecystitis (10)<br>Chronic cholecystitis (6)<br>Direct hyperbilirubinemia (obstructive jaundice) (16)<br>Transaminitis (2)	Notes: Study reported significantly higher incidence of cholelithiasis in SCD patients on<br>Hydroxyurea.<br>Limitations:<br>Patient adherence to Hydroxyurea treatment was not assessed.

					Acute pancreatitis (1)	
3	Al-Salem 1997	Cross sectional	Ages 8-18<br>SCD patients with CBD stones<br>N=18 (F-55.6%; M-45.4%)<br>Ethnicity Arab (Saudi-Arabia	Positive<br>sickling<br>test & Hb electrophore sis<br>SCD type unspecified	Etiology: Post-ERCP pancreatitis 5.6% (1)<br>Severity: NA<br>Complications: NA	Study focused on endoscopic and laparoscopic management of cholelithiasis and choledocholithiasis in children with SCD
4	Alwabari 2009	Cross sectional	Ages 2-15<br>SCD patients post-splenectomy or cholecystectom y<br>N=191 (Splenectomy group: F-40%; M-60%) (Cholecystecto my group: F-52.7%; M-47.3%)<br>Ethnicity - Arab (Saudi-Arabia)	HbSS<br>B-thalassemia major,<br>B-thalassemia intermediate	Etiology:<br>Biliary pancreatitis (N=1)<br>Severity: NA<br>Complications:<br>Acute chest syndrome, Postoperative fever, Bleeding from accessory cystic artery, Wound infection, Left pleural effusion in one, Bleeding at the splenic bed, Adhesive intestinal obstruction	The study compared safety & efficacy of open vs Laparoscopic splenectomy & cholecystectomy in<br>children with SCD<br>Only 1 child had pancreatitis
5	Amoako 2013	Cohort	Age <22<br>N=56 patients with 76 episodes of biliary tract	HbSS<br>HbSC	Etiology: Not specified<br>Severity: NA	Recurrent biliary tract disease is a frequent complication of SCD post-cholecystectomy

			disease. (7 patients with pancreatitis); 4 were secondary to obstruction from stone or sludge and 2 from CBD strictures<br>F=46.4%, M=53.6%	90% of episodes with HbSS, 9% in HbSC and 1% in sickle cell beta zero thalassemia	Complications:<br>Acute chest syndrome Bile peritonitis	Most patients (82% underwent cholecystectomy within 3 months of diagnosis with 50% recurrence rate by 6 months in those who did not undergo surgery.<br><br>The risk of recurrence was lower by 4 years after cholecystectomy. 13 episodes of biliary tract disease occurred after cholecystectomy. 15 of the recurrent episodes was treated with ERCP and 1 developed post ERCP pancreatitis
6	Banza 2019	Cross sectional	Ages 1-38 (Mean-11.8, SD-21.9)<br>SCD with Gall stone/ biliary & digestive diseases<br>(1 with Acute pancreatitis)<br>N=206 (F=51.9%, M=48.1%)<br><br>Ethnicity – African	Not specified	Etiology: Not specified<br>Association with BTD hypothesized<br><br>Severity: NA<br><br>Complications:<br>Splenomegaly	Hydroxyurea treatment<br><br>Digestive diseases are common in SCD patients

			Most represented ages 1-6 years (32.5% of cases)<br><br>43.68% (90) patients had no digestive complaints			
7	Bogue 2010	Cross sectional	Age<18<br>Children with Gallstones<br>N=382<br>(F=50%, M=50%)<br>Acute pancreatitis with choledocholithiasis (5) and 1 of whom had acute cholecystitis and choledocholithiasis<br><br>Ethnicity: NA (location of study – Canada)	Not specified	Etiology: Not specified<br><br>Severity: NA<br><br>Complications:<br>Choledocholithiasis (6), Pancreatitis (2), Acute cholecystitis (1), ERCP (3), Acute Renal Failure (1)	Management: Hospitalization, bowel rest, IVF, antibiotics, analgesia, cholecystectomy, and ERCP.<br><br>SCD & Spherocytosis are significant risk factors predisposing to symptomatic gallstones
8	Coats 2014	Cross sectional	Age range – Not specified<br>Mean age - 36.6 +/- 12.5 (SD)<br>N=767	HbSS – 62.7% (481)<br>HbSC – 31.8% (244)<br>Sickle beta 0 thalassemia (Hb S/β0-	Etiology: NA<br><br>Severity: NA<br><br>Serious complications: in 35 patients	Study found cholelithiasis in 43.8% (134) of SCD patients reviewed.---<br>HbSS: 88.8% (119)<br>HbSC : 9% (12)<br>HbSB+ : 1.5% (2)

			(F=57%, M=43%)<br><br>Ethnicity: NA (location of study – UK)	Thal) – 0.8% (6)<br>Sickle beta plus thalassemia (HbS β+thal) – 4.6% (35)<br>HbSHFPH – 0.1% (1)	AP: 5 patients<br>Acute cholangitis (4), Choledocholithiasis (8), Isolated cholecystitis (18)<br>Surgical complications in (3/34) who had cholecystectomy. Complications included bile leaks (2) and hepatic injury (1)	HbSB0: 0.7% (1)
9	Gale 2015	Cross sectional	Ages 1-18<br>Mean age (±SD) - 9.7±5.3 years<br><br>N=77<br>(No cholecystectomy - 52, Cholecystectomy - 25)<br>F=49.4%, M=50.6%<br><br>Ethnicity: NA (location of study – USA)	HbSS - 74% HbSC - 22.1 % HbSB+ - 2.6% HbSB0 - 1.3%	Etiology: NA<br><br>Severity: NA<br><br>Complications in 13/25 (52%) with cholecystectomy (11 before cholecystectomy and 2 after surgery- choledocholithiasis requiring sphincterotomy and stone retrieval and dropped stone with abscess formation)<br>Biliary obstruction (7) Intrahepatic biliary ductal	Pediatric SCD patients with cholelithiasis, other BTD are most likely to undergo cholecystectomy and associated complications.

					dilation, choledocholithiasis (4) (3 - requiring sphincterotomy and 1 - stone retrieval), Jaundice (2),<br><br>Gallstone pancreatitis (4)<br><br>Pathology-confirmed acute cholecystitis (1), dropped stone with abscess formation (N=1), and/or biliary dyskinesia (N= 1)	
10	Hanfy 2020	Retrospective	Age < 14 years of children with SCD admitted to PICU over a 6-year period<br><br>Location: Tabuk,Saudi Arabia<br><br>N= 58 patients with SCD admitted 93 times to the PICU<br><br>Median age: 9 years<br><br>Males: 33 (~57%)	HbSS: 52<br><br>HBS- Beta thalassemia - 6	SCD confirmed with Hb electrophoresis.<br><br>Majority of emergency admissions were for ACS: 28 (30%)<br><br>GI emergencies including pancreatitis, liver failure, hepatic sequestration accounted for 4 (4.59) (4.3%) of emergency total admissions	LOS positively correlated with WBC and CRP<br><br>27% of patients were on routine transfusions.<br><br>70% were on HU

11	Jasti 2008	Cross sectional	Age: Not specified<br><br>N=445<br>(<br>Ethnicity: NA (location of study – USA)	HbSS - 68.1% (303)<br>HbSC - 25.2% (112)<br>SCT (trait) - 6% HbS/β Thal trait - 0.7%<br><br>Only 7% (32) had AP<br>HbSS: 28<br>HbSC: 3<br>Sickle cell trait: 1	Etiology of AP in HbSS:<br>Gallstones – 71.4% (20)<br>Alcohol – 7.1% (2)<br>Small bowel obstruction/ Tylenol toxicity – 3.6% (1 each)<br>Idiopathic - 14.3% (4)<br><br>Etiology of AP in HbSC: Gallstones - 66.7% (2)<br>Idiopathic - 33.3% (1)<br><br>Etiology of AP in SCT: Colonic perforation from diverticulitis – 3.6%<br><br>Complication:<br>Death attributed to AP occurred in one HbSS patient (3%)	Prevalence of AP in SCD is similar to general population and may result in death.<br><br>Limitation: Data only available for those who were tested
12	Nourallah 1998	Cross sectional	Ages 8-18<br>SCD patients with diagnostic & therapeutic ERCP	Not specified	Etiology: NA<br>Indications for ERCP:	Conservative treatment

			N=24<br>(F=37.5%, (9)M=62.5%) (15)<br>Ethnicity: Arab		Obstructive jaundice – 75% (18)<br>Pancreatitis – 8.3% (2)<br>Cholangitis – 8.3% (2)<br>Recurrent biliary colic – 4.2% (1)<br>Post-laparoscopic cholecystectomy with bile leak – 4.2% (1)	
13	Sakhalkar 2004		Children's age range Not specified.<br>SCD with AP<br>N=500<br>Ethnicity: Not specified (study location – USA)	HbSC - 33% HbSB+ Thal - 2-4%<br>others<br>HbSS<br>HbSB0	Etiology: NA<br>Severity: NA<br>Complications:<br>Edema of CBD, Acute Chest Syndrome, Sepsis, CBD stricture	
14	Sharma 2019	Cross sectional	Age – Not specified<br>SCD patients with & without *Clostridioides difficile* infection (CDI)<br>N=747,127 hospitalized patients with SCD	Not specified	Etiology: NA<br>Severity: NA<br>Complications:<br>Acute chest syndrome (6.2%), splenic sequestration (1.9%), and	African Americans hospitalized with SCD, other BTD, including AP, chronic pancreatitis were more likely to have CDI.<br>Associated with increasing mortality

			Ethnicity: Not specified (study location – USA)<br><br>804 had SCD + CDI		vaso-occlusive crises (4.1%)	
15	Ziegler 1988	Cross sectional	Ages 1 month-18 years<br><br>Pediatric patients with Pancreatitis<br>N=49<br>(F=44.9%, M=55.1%)<br><br>Ethnicity: Not specified (study location – USA)<br><br>AP diagnosed with elevated serum/ urine amylase	Not specified	Etiology of AP:<br>BTD – 32.7% (16) (SCA-12.3%) (6)<br>Trauma – 33% (non motorized vehicle in 5 cases while MVA in 3)<br>10% (5)- Child abuse<br>Other blunt trauma caused 3 cases<br><br>Severity: NA<br><br>Complications:<br>Pseudocysts, Pancreatic abscesses, Sepsis,<br>Respiratory failure, Renal failure	IVF, Nasogastric suction, TPN

Key: BTD – Biliary tract disease, SCT – Sickle-cell trait
